# Prevalence of human respiratory pathogens and associated mucosal cytokine levels in young children and adults: a cross-sectional observational study in the Netherlands during the winter of 2012/2013

**DOI:** 10.1093/femspd/ftae010

**Published:** 2024-05-07

**Authors:** Puck B van Kasteren, Anne T Gelderloos, Mioara Alina Nicolaie, Gerco den Hartog, Marloes Vissers, Willem Luytjes, Nynke Y Rots, Josine van Beek

**Affiliations:** Center for Infectious Disease Control, National Institute for Public Health and the Environment, Antonie van Leeuwenhoeklaan 9, 3721 MA, Bilthoven, The Netherlands; Center for Infectious Disease Control, National Institute for Public Health and the Environment, Antonie van Leeuwenhoeklaan 9, 3721 MA, Bilthoven, The Netherlands; Center for Infectious Disease Control, National Institute for Public Health and the Environment, Antonie van Leeuwenhoeklaan 9, 3721 MA, Bilthoven, The Netherlands; Center for Infectious Disease Control, National Institute for Public Health and the Environment, Antonie van Leeuwenhoeklaan 9, 3721 MA, Bilthoven, The Netherlands; Center for Infectious Disease Control, National Institute for Public Health and the Environment, Antonie van Leeuwenhoeklaan 9, 3721 MA, Bilthoven, The Netherlands; Center for Infectious Disease Control, National Institute for Public Health and the Environment, Antonie van Leeuwenhoeklaan 9, 3721 MA, Bilthoven, The Netherlands; Center for Infectious Disease Control, National Institute for Public Health and the Environment, Antonie van Leeuwenhoeklaan 9, 3721 MA, Bilthoven, The Netherlands; Center for Infectious Disease Control, National Institute for Public Health and the Environment, Antonie van Leeuwenhoeklaan 9, 3721 MA, Bilthoven, The Netherlands

**Keywords:** viral interference, mucosal immunity, inflammation, coinfection, ageing, infants

## Abstract

Respiratory pathogens can cause severe disease and even death, especially in the very young and very old. Studies investigating their prevalence often focus on individuals presenting to healthcare providers with symptoms. However, the design of prevention strategies, e.g. which target groups to vaccinate, will benefit from knowledge on the prevalence of, risk factors for and host response to these pathogens in the general population. In this study, upper respiratory samples (*n* = 1311) were collected cross-sectionally during winter from 11- and 24-month old children, their parents, and adults ≥60 years of age that were recruited irrespective of seeking medical care. Almost all children, approximately two-thirds of parents and a quarter of older adults tested positive for at least one pathogen, often in the absence of symptoms. Viral interference was evident for the combination of rhinovirus and respiratory syncytial virus. Attending childcare facilities and having siblings associated with increased pathogen counts in children. On average, children showed increased levels of mucosal cytokines compared to parents and especially proinflammatory molecules associated with the presence of symptoms. These findings may guide further research into transmission patterns of respiratory pathogens and assist in determining the most appropriate strategies for the prediction and prevention of disease.

## Teaser

Respiratory pathogen prevalence in the general population differs between young children and (older) adults and nasal proinflammatory cytokine concentrations associate with the presence of (mild) symptoms of respiratory infection.

## Introduction

Respiratory infections are a major cause of morbidity and mortality, most prominently in the very young and very old. Whereas SARS-CoV-2 has illustrated the havoc a respiratory pathogen can wreak during a pandemic, endemic respiratory pathogens such as influenza virus, *Streptococcus pneumoniae*, and respiratory syncytial virus (RSV) have continuously posed a considerable threat to public health in the past, and will likely do so in the future (PERCH Study Group 2019, Shi et al. 2019). A large number of human pathogens that target the respiratory tract are known, some of which can readily cause severe disease while others mostly do not (Sarna et al. 2018, PERCH Study Group 2019). Licensed vaccines are presently only available for a small number of these pathogens, including influenza virus, RSV, *S. pneumoniae*, and *Haemophilus influenzae*.

For knowledge-based decision making on the implementation of preventative measures such as risk group-specific vaccination against endemic respiratory pathogens, a thorough understanding of their prevalence in the general population is needed. However, the majority of studies relating to the occurrence of respiratory pathogens focus on medically attended infections. While these studies are important for estimating the burden of disease and determining which groups are at risk for severe illness, they mostly do not provide information on the general presence of these pathogens in the population. Collecting such data requires active sampling of mostly healthy participants from various subpopulations and the use of broad diagnostic panels, which is not often undertaken or, when they are, often focus primarily on infants (Jansen et al. 2011, Byington et al. 2015, Moe et al. 2016, Birger et al. 2018, Sarna et al. 2018, Galanti et al. 2019, Zoch-Lesniak et al. 2020, Powell et al. 2022).

In addition to population-based prevention of respiratory infections, morbidity and mortality might be decreased by the early identification of infected individuals that are likely to develop severe disease. Cytokines and chemokines are important mediators of the immune response and are therefore potential biomarkers or even determinants for severe illness (Rot and von Andrian 2004, Garcia et al. 2012, Bohmwald et al. 2019, Glaser et al. 2019). Proper interpretation of the role of these molecules in the development of severe disease will likely benefit from increased knowledge regarding their baseline presence during mild or asymptomatic infections (van Woudenbergh et al. 2023).

In this cross-sectional study performed in the Netherlands during the winter months, we have assessed the prevalence in the upper respiratory mucosa of a wide variety of respiratory pathogens, both viral and bacterial, in different age groups and in the absence of serious illness. In addition, we have assessed childcare and having siblings as risk factors associated with higher pathogen counts in young children. Finally, we determined the concentration in nasopharyngeal swab samples of a range of (proinflammatory) cytokines/chemokines and assessed their association with symptoms of respiratory infection.

## Materials and methods

### Study design

Upper respiratory swab samples, both nasopharyngeal and oropharyngeal (adults only), were collected as part of two cross-sectional, observational cohort studies, which were performed throughout the winter of 2012/2013 in the Netherlands. For the exact timing of sampling, see Supplementary Fig. S1A. The studies were designed and run in parallel to allow for combined analysis. Study A was primarily designed to assess carriage of *S. pneumoniae* in children and adults in relation to changes in the national immunization program (Bosch et al. 2016). Study B was primarily designed to identify the causative agents of influenza-like illness in community-dwelling older adults (van Beek et al. 2017). Study participants belonged to one of four groups: 11-month old children (*n* = 327, study A), 24-month old children (*n* = 325, study A), parents of the 24-month old children (*n* = 319, study A), and older adults ≥60 years of age (*n* = 340, study B). Exclusion criteria for study A were known or suspected immunodeficiency, craniofacial or chromosomal abnormalities, and coagulation disorders or use of anticoagulant medication. There were no exclusion criteria for study B. However, the present analysis includes only the baseline controls of study B and therefore does not include participants presenting with fever at the time of sampling, as these would have been designated as cases according to the study protocol. Throughout the study period, trained research nurses performed scheduled home visits that consisted of a structured interview and sample collection. Each participant was sampled once during the entire study period.

### Interview

By means of a structured interview during the scheduled home visit data was obtained regarding age, sex, body-mass index (BMI; adults only), breastfeeding history (children only), childcare attendance (children only), and having siblings (children only). In addition, participants were asked the following question during the interview (translated from Dutch): are you at this moment experiencing a common cold and/or do you have complaints of a respiratory infection, such as otitis, laryngitis, sinusitis, bronchitis, or pneumonia? Parents were asked to answer the questions for their child.

### Sampling

Nasopharyngeal (all groups) and oropharyngeal (adults only) samples were obtained according to World Health Organization standard procedures with a sterile swab with a flocked nylon tip and stored in 1 ml modified liquid Amies transport medium (Eswab; Copan, Brescia, Italy). Swab samples were transported at room temperature to the laboratory, where the samples were aliquoted and subsequently used within 12 hours after sampling for bacterial culturing or stored at −80°C within 8 hours after sampling for subsequent viral diagnostics and cytokine analysis. Sample collection and processing were performed using the same protocols and by the same teams of research nurses and laboratory personnel for both studies.

### Pathogen diagnostics

Viral diagnostics were performed for all individuals on nucleic acids isolated from nasopharyngeal swabs (easyMAG, BioMérieux) by a multiplex ligation-dependent probe amplification assay for a broad panel of respiratory viruses, including influenza A and B viruses, RSV A and B, human metapneumovirus (hMPV), rhino/enterovirus, adenovirus (AdV), parainfluenza viruses (PIV) 1 to 4, human bocavirus (hBoV), and human coronavirus (hCoV) NL63, OC43, 229E, and HKU1 (RespiFinder Smart 22; PathoFinder, Maastricht, the Netherlands). In addition, Karolinska Institute polyomavirus (KIPyV), Washington University polyomavirus (WUPyV), and influenza virus subtypes (H1N1, H3N2, Yamagata, Victoria) were determined by reverse transcriptase PCR as described previously (Bialasiewicz et al. 2007, Meijer et al. 2009). The presence of *S. pneumoniae, H. influenzae, Moraxella catarrhalis*, and *Staphylococcus aureus* in nasopharyngeal (children and parents) and oropharyngeal (parents and older adults) swab samples was determined by standard diagnostic culturing procedures.

### Multiplex immunoassay for cytokines

The concentrations of a collection of cytokines and chemokines were determined in a selection of nasopharyngeal swab samples from 11- and 24-month old children and parents using a multiplex immunoassay (LegendPlex, BioLegend) according to the manufacturer’s instructions. Samples had been stored at −80°C for 5 years prior to the analysis. From each group, ~100 individuals were randomly selected while maintaining equal female to male ratios. To assess the local inflammatory profile, the following panels were measured: “human proinflammatory chemokine panel” (CXCL1, CXCL5, CXCL8, CXCL9, CXCL10, CXCL11, CCL2, CCL3, CCL4, CCL5, CCL11, CCL17, and CCL20), “human inflammation panel 1” (IL-1β, IL-6, IL-10, IL-12p70, IL-17A, IL-18, IL-23, IL-33, IFN-α2, IFN-γ, TNF α, CXCL8, and CCL2), and a custom panel consisting of IL-4, IL-13, IL-1Rα, GM-CSF, IFN-β, granzyme A, granzyme B, perforin, and granulysin. Of these cytokines, IL-17A, IL-4, IL-1Rα, GM-CSF, and perforin were excluded from further analysis because a large proportion of measurements was below the detection limit. For analytical purposes, individual measurements that were below the detection limit were assigned a value of 0.5 times the lowest concentration that had been determined for that specific target.

### Statistical analysis and visualization

Comparisons between categorical data were assessed using the Fisher’s exact test. Cytokine concentrations for group comparisons are presented in boxplots with median and quartiles while showing individual data points. Differences in cytokine concentrations or pathogen counts between groups were assessed using a nonparametric Kruskal–Wallis test, followed by Dunn’s multiple comparisons test. Correlations were assessed using the Spearman rank order method. Interaction of risk factors for numerical data was assessed using a Kruskal–Wallis test and for categorical data using a generalized Cochran–Mantel–Haenszel test, where for both tests the *P*-value was evaluated by permutation test.

Statistical analysis and graph design were performed with GraphPad Prism 9.3.1 software or R Statistical Software v4.3.0 and Rstudio v2023.03.1. The cytokine heatmap was generated with log10-transformed data using the pheatmap package v1.0.12 in which the Euclidean method was used to calculate distance and complete linkage clustering was performed on both rows and columns. The cytokine correlation matrix was generated based on Spearman rank order correlation using the rcorr function in the Hmisc package v5.1–1.

## Results

### Description of study population, sampling time, and diagnostics

To assess the prevalence of respiratory pathogens in the general population, a study cohort consisting of 11- and 24-month old children, their parents, and older adults ≥60 years of age was formed. Participant demographics can be found in Table 1. Each group consisted of 319–340 participants, with approximately equal female to male ratios except for parents where 82% of participants was female. The mean age for parents and older adults was 35 years and 74 years, respectively. Breastfeeding history, childcare attendance, and having siblings was comparable between 11- and 24-month old children. Of the older adults, 77% had received an influenza vaccination in 2012, while this was ~7% for parents. In the Netherlands, older adults of ≥60 years and individuals with specific medical conditions are eligible for influenza vaccination within the national immunization program. Dutch children do not routinely receive influenza vaccination as part of the national immunization program.

**Table 1.** Participant characteristics, total study population.

	11-months	24-months	Parents^a^	Older adults
Sample size, *n*	327	325	319	340
Female sex, number (%)	153 (46.8)	181 (55.7)	261 (81.8)	185 (54.4)
Age in days, mean (range)	329 (308–362)	737 (701–759)		
Age in years, mean (range)			35 (22–51)	74 (61–92)
**Breastfeeding history** **^b^**				
Never, number (%)	60 (18.3)	65 (20.0)		
0 < 6 months, number (%)	126 (38.5)	121 (37.3)		
≥6 months, number (%)	141 (43.1)	138 (42.6)		
**Childcare**				
≥0.5 days/week, number (%)	235 (71.9)	251 (77.2)		
Duration in days/week, mean (range)^c^	2.1 (0.5–5)	2.2 (0.5–5)		
Group size, mean (range)	9.3 (0–18)	10 (0–30)		
**Siblings**				
≥1 siblings, number (%)	182 (55.7)	204 (62.8)		
Number of siblings, mean (range)^d^	1.3 (1–3)	1.4 (1–4)		
**BMI** **^e^**				
Mean (range)			24.7 (16.9–44.1)	25.8 (16.4–39.8)
≥25, number (%)			118 (37.0)	189 (55.9)
**Influenza vaccination**				
2012/2013, number (%)			23 (7.2)	261 (76.8)

a Parents of the 24-month old children.

b Data are missing for one child in the 24 months group.

c For those children that attend childcare facilities.

d For those children that have siblings.

e Data are missing for two older adult participants.

One sampling moment was randomly scheduled for each study participant during the winter months (October–March) of 2012/2013, for an overview see Supplementary Fig. S1(A). Pathogen diagnostics were performed on upper respiratory swab samples to determine the presence of influenza viruses, RSV, hMPV, rhino/enterovirus, AdV, PIV, hBoV, KIPyV, WUPyV, seasonal hCoV, *S. pneumoniae, H. influenzae, M. catarrhalis*, and *S. aureus*. Diagnostics data concerning viruses in older adults and bacteria in children/parents have been published before (Bosch et al. 2016, van Beek et al. 2017), but are reproduced here in more detail for comparative purposes. Virus diagnostics were performed on nasopharyngeal swab samples for all groups. Bacterial diagnostic culturing was performed on nasopharyngeal swab samples for children and parents, and on oropharyngeal swab samples for parents and older adults.

### Children and adults show differing patterns of viral and bacterial prevalence in the upper respiratory mucosa

**Figure 1.**
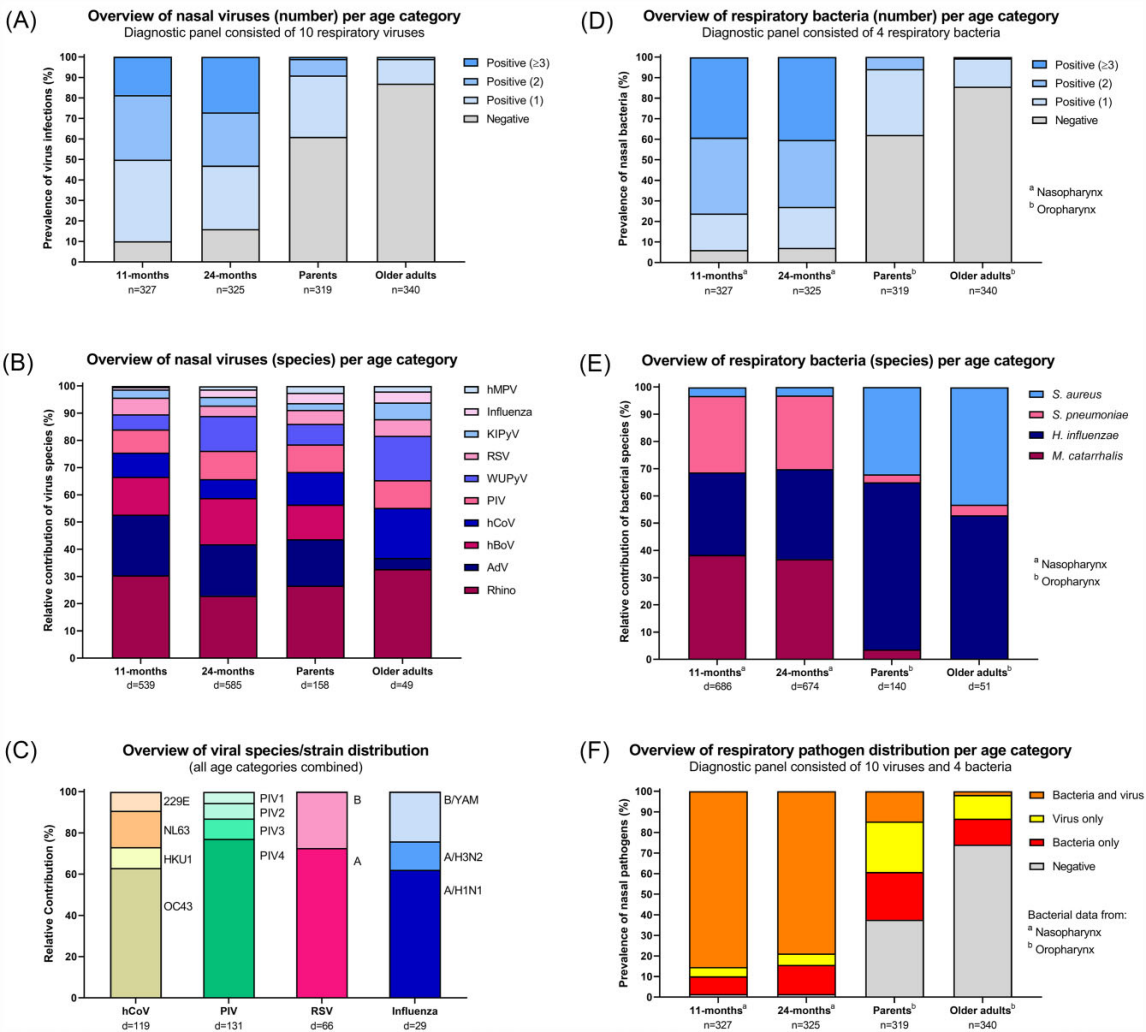
Detection of pathogens in the respiratory mucosa in different age groups. (A) The prevalence of respiratory viral infections in all participants (*n* indicates group size) and (B) the relative contribution of viral species (d indicates the total number of individual pathogen detections, a single participant can be included multiple times with the occurrence of codetections) in different age groups. (C) Further subdivision in species/subtypes for endemic human CoVs, PIV, RSV, and influenza virus for all age groups combined. (D) The prevalence of respiratory bacteria and (E) the relative contribution of bacterial species in different age groups. (F) Overview of the distribution of viral infections and bacteria between individuals in different age groups. Groups consisted of 11-month old children (*n* = 327), 24-month old children (*n* = 325), parents (*n* = 319), and older adults ≥60 years of age (*n* = 340). Bacterial presence was determined in (a) nasopharyngeal or (b) oropharyngeal swabs. Abbreviations: AdV, adenovirus; hBoV, human bocavirus; hCoV, human coronavirus; hMPV, human metapneumovirus; KIPyV, Karolinska Institute polyomavirus; Rhino, rhinovirus; RSV, respiratory syncytial virus; PIV, parainfluenza virus; and WUPyV, Washington University polyomavirus. Data concerning virus detections in older adults and bacteria in children/parents have been previously described in less detail and are shown here for comparative purposes (Bosch et al. 2016, van Beek et al. 2017).

We first assessed the prevalence of viral pathogens per age group, irrespective of viral species (Fig. 1A). Notably, in the 11-month old age group only 33 out of 327 children (10%) were negative for all of the viruses tested, while ~50% was positive for at least two (and up to five) different viruses. In the 24-month old age group, slightly more children tested negative for viral infections (52/325 or 16%), but still approximately half were positive for at least two (and up to six) different viruses. In contrast, in older adults the vast majority (87%) of individuals tested negative for viral infections and the occurrence of viral codetections was rare (1%). The parent group showed an intermediate profile with 61% testing negative for viral infection and 9% of individuals displaying viral codetections.

Next, we assessed whether certain virus species are relatively more common compared to other species in specific age groups. For this, we calculated the relative contribution of each virus to the total number of virus detections per group (Fig. 1B). Of note, each individual potentially contributes more than one count to the total number of virus detections depending on the presence of codetections. From these data, it is clear that especially hBoV, but also adenovirus, are relatively more common in children and parents compared to older adults. In contrast, endemic hCoVs appear to be relatively more common in older adults, compared to children and parents. Interestingly, WU polyomavirus displayed the highest relative contributions in older adults and 24-month old children.

For some viruses, more detailed classification of the exact viral species or strains was performed (Fig. 1C). Data combined for all study participants showed that of the four endemic coronaviruses circulating at that time, OC43 was relatively most abundant (63%), followed at a distance by NL63 (18%). Furthermore, PIV 4 (77%), RSV A (73%), and influenza virus A/H1N1 (62%), followed by B/YAM (24%), were the most abundant strains/species in their respective virus groups.

The prevalence of bacterial pathogens per age group, irrespective of species (Fig. 1D), showed a similar pattern as the viral pathogen prevalence. The vast majority of children tested positive for bacterial pathogens (>92% in both groups) and bacterial codetections were frequent (>72% for both groups) in nasopharyngeal swabs. The majority of older adults (86%) tested negative for bacterial pathogens and bacterial codetections were rarely seen (<1%) in oropharyngeal swabs. Again, parents showed an intermediate profile with 62% testing negative and 6% showing bacterial codetection in oropharyngeal swabs, with a slightly higher bacterial prevalence in nasopharyngeal swabs (Supplementary Fig. S1B). When looking at bacterial species specifically, it is clear that *S. aureus* was relatively more abundant in adults than in children, while *S. pneumoniae* and *M. catarrhalis* were relatively more abundant in children than in adults (Fig. 1E). The apparent difference in *H. influenzae* prevalence between children and adults appears to reflect the different sampling sites, as parental oropharyngeal swabs show a considerably higher relative contribution of this species than nasopharyngeal swabs (Supplementary Fig. S1C).

Next, we were interested in the distribution of viral and bacterial pathogens between individuals (Fig. 1F). As could be expected from the individual viral and bacterial prevalence data, the simultaneous presence of both viral and bacterial pathogens was most frequently and indeed quite often observed among children (85% and 79% for 11-month old children and 24-month old children, respectively) and children that tested negative for all pathogens were very rare (1.5% for both groups). For older adults only a small proportion of individuals was simultaneously positive for both viral and bacterial pathogens (<2%) and this proportion was higher for parents (15%). Furthermore, 74% of older adults and 38% of parents tested negative for all pathogens. Nasopharyngeal and oropharyngeal swab bacterial data yielded largely similar results for the pathogen distribution pattern in parents (Supplementary Fig. S1D).

### Several pathogen combinations occur at a different frequency than would be expected based on individual pathogen prevalence

Considering previous reports on pathogen interference (Piret and Boivin 2022, Takashima et al. 2022), we used the diagnostics data from children to analyze the frequency of codetections between different pathogen species (Table 2). In line with other reports (Achten et al. 2017, Wu et al. 2020, Takashima et al. 2022), we observed that rhinoviruses appear to be less frequently detected in combination with RSV, hMPV, influenza virus, AdV, hCoV, and WUPyV than expected based on their frequency in the total study population. However, our sample only supports evidence for the combination of rhinovirus with RSV (corrected *P* = .019, Fisher’s exact test, FDR <20%) and WUPyV (corrected *P* = .17, FDR <20%). In addition, our sample supports evidence for less frequent codetection than expected for the combination hBoV with hCoV (corrected *P* = .15, FDR <20%). In contrast, PIV, hBoV, and WUPyV are more often detected in combination with each other than expected, as is the combination of PIV with KIPyV. Regarding bacterial codetections, *S. aureus* is detected together with *M. catarrhalis, H. influenzae*, and *S. pneumoniae* less frequently than expected. In contrast, *M. catarrhalis, H. influenzae*, and *S. pneumoniae* are more often detected in combination with each other than expected. Finally, the combination of AdV with *M. catarrhalis* or *H. influenzae* occurs more frequently than expected. Corrected *P*-values and odds ratios for all codetection pairs with an FDR <20% can be found in Table 3.

**Table 2.** Pathogen codetections in 11- and 24-month old children combined.

*n* = 652	Rhino	AdV	hBoV	hCoV	PIV	RSV	hMPV	WUPyV	KIPyV	Influenza	*M. cat*	*H. inf*	*S. pneu*	*S. aureus*
*n* (%)	298 (45.7)	231 (35.4)	174 (26.7)	89 (13.7)	107 (16.4)	55 (8.4)	9 (1.4)	105 (16.1)	35 (5.4)	21 (3.2)	511 (78.4)	431 (66.1)	375 (57.5)	43 (6.6)
Rhino	7 (2.3)	94 (31.5)	83 (27.9)	36 (12.1)	50 (16.8)	**14 (4.7)**	2 (0.7)	**38 (12.8)**	18 (6.0)	5 (1.7)	243 (81.5)	196 (65.8)	181 (60.7)	21 (7.0)
AdV	94 (40.7)	4 (1.7)	72 (31.2)	34 (14.7)	44 (19.0)	17 (7.4)	2 (0.9)	44 (19.0)	17 (7.4)	8 (3.5)	**196 (84.8)**	**170 (73.6)**	138 (59.7)	**8 (3.5)**
hBoV	83 (47.7)	72 (41.4)	1 (0.6)	**15 (8.6)**	**55 (31.6)**	19 (10.9)	**6 (3.4)**	**45 (25.9)**	13 (7.5)	6 (3.4)	144 (82.8)	125 (71.8)	102 (58.6)	9 (5.2)
hCoV	36 (40.4)	34 (38.2)	**15 (16.9)**	1 (1.1)	10 (11.2)	5 (5.6)	0 (0.0)	14 (15.7)	8 (9.0)	2 (2.2)	77 (86.5)	63 (70.8)	59 (66.3)	3 (3.4)
PIV	50 (46.7)	44 (41.1)	**55 (51.4)**	10 (9.3)	2 (1.9)	11 (10.3)	2 (1.9)	**27 (25.2)**	**13 (12.1)**	4 (3.7)	87 (81.3)	77 (72.0)	67 (62.6)	5 (4.7)
RSV	**14 (25.5)**	17 (30.9)	19 (34.5)	5 (9.1)	11 (20.0)	1 (1.8)	0 (0.0)	9 (16.4)	5 (9.1)	0 (0.0)	47 (85.5)	38 (69.1)	35 (63.6)	5 (9.1)
hMPV	2 (22.2)	2 (22.2)	**6 (66.7)**	0 (0.0)	2 (22.2)	0 (0.0)	0 (0.0)	3 (33.3)	0 (0.0)	0 (0.0)	7 (77.8)	5 (55.6)	8 (88.9)	2 (22.2)
WUPyV	**38 (36.2)**	44 (41.9)	**45 (42.9)**	14 (13.3)	**27 (25.7)**	9 (8.6)	3 (2.9)	5 (4.8)	5 (4.8)	5 (4.8)	85 (81.0)	77 (73.3)	64 (61.0)	6 (5.7)
KIPyV	18 (51.4)	17 (48.6)	13 (37.1)	8 (22.9)	**13 (37.1)**	5 (14.3)	0 (0.0)	5 (14.3)	0 (0.0)	2 (5.7)	**33 (94.3)**	25 (71.4)	21 (60.0)	2 (5.7)
Influenza	5 (23.8)	8 (38.1)	6 (28.6)	2 (9.5)	4 (19.0)	0 (0.0)	0 (0.0)	5 (23.8)	2 (9.5)	0 (0.0)	14 (66.7)	18 (85.7)	11 (52.4)	0 (0.0)
*M. cat*	243 (47.6)	**196 (38.4)**	144 (28.2)	77 (15.1)	87 (17.0)	47 (9.2)	7 (1.4)	85 (16.6)	**33 (6.5)**	14 (2.7)	13 (2.5)	**363 (71.0)**	**328 (64.2)**	**23 (4.5)**
*H. inf*	196 (45.5)	**170 (39.4)**	125 (29.0)	63 (14.6)	77 (17.9)	38 (8.8)	5 (1.2)	77 (17.9)	25 (5.8)	18 (4.2)	**363 (84.2)**	8 (1.9)	**275 (63.8)**	**17 (3.9)**
*S. pneu*	181 (48.3)	138 (36.8)	102 (27.2)	59 (15.7)	67 (17.9)	35 (9.3)	8 (2.1)	64 (17.1)	21 (5.6)	11 (2.9)	**328 (87.5)**	**275 (73.3)**	4 (1.1)	**16 (4.3)**
*S. aureus*	21 (48.8)	**8 (18.6)**	9 (20.9)	3 (7.0)	5 (11.6)	5 (11.6)	2 (4.7)	6 (14.0)	2 (4.7)	0 (0.0)	**23 (53.5)**	**17 (39.5)**	**16 (37.2)**	2 (4.7)

Guide for interpretation: cells show the number of codetections of column and row pathogen, with the detection frequency of the column pathogen as a percentage of the total number of row pathogen detections between brackets. For example, in 94 children both rhinovirus and adenovirus were detected. Since there were a total of 231 children positive for adenovirus (*n* in top row below AdV), this means that 40.7% (94/231 × 100%) of adenovirus-infected children also had a rhinovirus infection. As can be observed in the top row of the rhinovirus column, of all children (*n* = 652) there were 298 (45.7%) positive for rhinovirus infection. This latter percentage is thus the frequency of rhinovirus infection in the total population under study, which is regarded as the “expected” frequency of codetections with rhinovirus in the absence of pathogen interactions. Notably, a child that is infected with rhinovirus, adenovirus, and bocavirus will be counted twice in the rhinovirus column, which is the reason that the sum of counts per column is higher than the total number of children infected with the column pathogen. Codetection pairs for which the frequency is significantly different from what is expected (Fisher’s exact test, with a correction for multiple testing at a false discovery rate <20%) are indicated in bold, also see Table 3. Cells where column and row pathogen are identical indicate the number of children that were positive for this pathogen alone. For example, there were seven children that tested positive for only rhinovirus, which was 2.3% of all rhinovirus infections. Abbreviations: Rhino, rhinovirus; AdV, adenovirus; hBoV, human bocavirus; hCoV, human coronavirus; PIV, parainfluenza virus; RSV, respiratory syncytial virus; hMPV, human metapneumovirus; WUPyV, Washington University polyomavirus; KIPyV, Karolinska Institute polyomavirus; *M. cat, Moraxella catarrhalis; H. inf, Haemophilus influenzae; S. pneu, Streptococcus pneumoniae*; and *S. aureus, Staphylococcus aureus*.

**Table 3.** List of codetected pathogen pairs in children for which the observed frequency differs from the expected frequency at a FDR <20%.

Pathogens		Counts	Odds ratio (range)	*P*-value	Corrected *P*-value^a^
hBoV	hMPV	6	5.62 (1.19–35.1)	.014	.088
KIPyV	*M. cat*	33	4.79 (1.20–41.7)	.018	.11
hBoV	PIV	55	3.77 (2.40–5.94)	<.001	<.001
*S. pneu*	*M. cat*	328	3.58 (2.38–5.44)	<.001	<.001
PIV	KIPyV	13	3.28 (1.46–7.08)	.002	.019
*H. inf*	*M. cat*	363	2.63 (1.76–3.93)	<.001	<.001
hBoV	WUPyV	45	2.42 (1.53–3.82)	<.001	.002
*S. pneu*	*H. inf*	275	2.13 (1.51–3.01)	<.001	<.001
PIV	WUPyV	27	2.02 (1.18–3.39)	.009	.063
AdV	*M. cat*	196	1.88 (1.22–2.96)	.003	.026
AdV	*H. inf*	170	1.71 (1.19–2.48)	.003	.027
Rhino	WUPyV	38	0.62 (0.39–0.98)	.033	.17
hCoV	hBoV	15	0.51 (0.27–0.94)	.028	.15
*S. aureus*	*S. pneu*	16	0.41 (0.20–0.81)	.006	.049
*S. aureus*	AdV	8	0.40 (0.16–0.89)	.020	.11
Rhino	RSV	14	0.38 (0.19–0.72)	.002	.019
*S. aureus*	*H. inf*	17	0.31 (0.15–0.61)	<.001	.004
*S. aureus*	*M. cat*	23	0.29 (0.14–0.57)	<.001	.003

a Fisher’s exact test, with a correction for multiple testing at a false discovery rate (FDR) <20%.

Abbreviations: Rhino, rhinovirus; AdV, adenovirus; hBoV, human bocavirus; hCoV, human seasonal coronaviruses; PIV, parainfluenza virus; RSV, respiratory syncytial virus; hMPV, human metapneumovirus; WUPyV, Washington University polyomavirus; *M. cat, Moraxella catarrhalis; H. inf, Haemophilus influenzae; S. aureus, Staphylococcus aureus; S. pneu, Streptococcus pneumoniae*.

### Childcare attendance and having siblings strongly associate with higher pathogen counts in children

Having established that the presence of multiple pathogens simultaneously in the nasopharyngeal mucosa is quite common in children, we were interested in the factors that potentially contribute to the occurrence of such compounded detections. Based on the information obtained from the interview that was performed during the home visits, we assessed the association of infection status with childcare attendance and having siblings (Table 4 and Supplementary Fig. S2A–D). We found that both 11- and 24-month old children that attended childcare facilities (≥4 hours per week) had a significantly higher mean number of pathogen species in their nasopharyngeal mucosa than children who did not attend childcare facilities (*P* < .001, Kruskall–Wallis with Dunn’s multiple comparisons test). Of note, none of the children that tested negative for all pathogens (*n* = 10) attended childcare facilities. Similarly, children that attended childcare were significantly more likely to test positive for both bacterial and viral pathogens simultaneously than children that did not attend childcare facilities (*P* < .001, Fisher’s exact test). Furthermore, we found that 11-month old children, but not 24-month old children, with at least one sibling had a significantly higher mean number of pathogens in their nasopharyngeal mucosa than children without siblings (*P* = .004, Kruskal–Wallis). In addition, 11-month old children with siblings were significantly more likely to test positive for both bacterial and viral pathogens simultaneously than children without siblings (*P* = .003, Fisher’s exact test). Finally, the combination of childcare and having siblings in 11-month old children appears to be an even more prominent risk factor than either of the two factors by itself (Supplementary  Fig. S2E and F), for both pathogen counts (*P* < .001, Kruskall–Wallis) and simultaneous detection of bacteria and viruses (*P* < .001, generalized Cochran–Mantel–Haenszel Test).

**Table 4.** Risk factors for infection in children and adults, univariate analysis.

Childcare attendance	No	Yes	OR (95% CI)	*P-*value
*11-months*				
Pathogen count, mean (95% CI)	2.7 (2.4–3.0)	4.2 (4.0–4.3)	na	<.001^1^
Bacterial and viral codetections, number (%)	62/92 (67%)	217/235 (92%)	5.8 (3.1–11)	<.001^2^
*24-months*				
Pathogen count, mean (95% CI)	2.8 (2.4–3.1)	4.2 (4.0–4.4)	na	<.001^1^
Bacterial and viral codetections, number (%)	42/74 (57%)	214/251 (85%)	4.4 (2.5–7.8)	<.001^2^
**Having siblings**	**No**	**Yes**		***P-*value**
*11-months*				
Pathogen count, mean (95% CI)	3.4 (3.2–3.7)	4.0 (3.8–4.2)	na	.004^1^
Bacterial and viral codetections, number (%)	114/145 (79%)	165/182 (91%)	2.6 (1.4–5.0)	.003^2^
*24-months*				
Pathogen count, mean (95% CI)	3.9 (3.6–4.2)	3.9 (3.6–4.1)	na	ns^1^
Bacterial and viral codetections, number (%)	98/121 (81%)	158/204 (77%)	0.8 (0.5–1.4)	ns^2^

1 Kruskall–Wallis with Dunn’s multiple comparisons test.

2 Fisher’s exact test with Baptista–Pike for odds ratio CI.

Abbreviations: CI, confidence interval; na, not applicable; and OR, odds ratio.

As already apparent from Fig. 1(A) and (D), considerable differences exist in the prevalence of respiratory pathogens between parents and older adults. Parents had on average both more viruses and more bacteria in their upper respiratory tract than older adults (*P* < .001, Kruskal–Wallis; Supplementary  Fig. S2G). In addition, parents were significantly more likely to test positive both for viruses and for bacteria than older adults (*P* < .001, Fisher’s exact test; Supplementary  Fig. S2H). Finally, we assessed whether sex or a BMI ≥25 associated with infection status in parents, but no differences were apparent (Supplementary Fig. S2I and J).

### The prevalence of symptoms of respiratory infection differs between age groups

**Figure 2.**
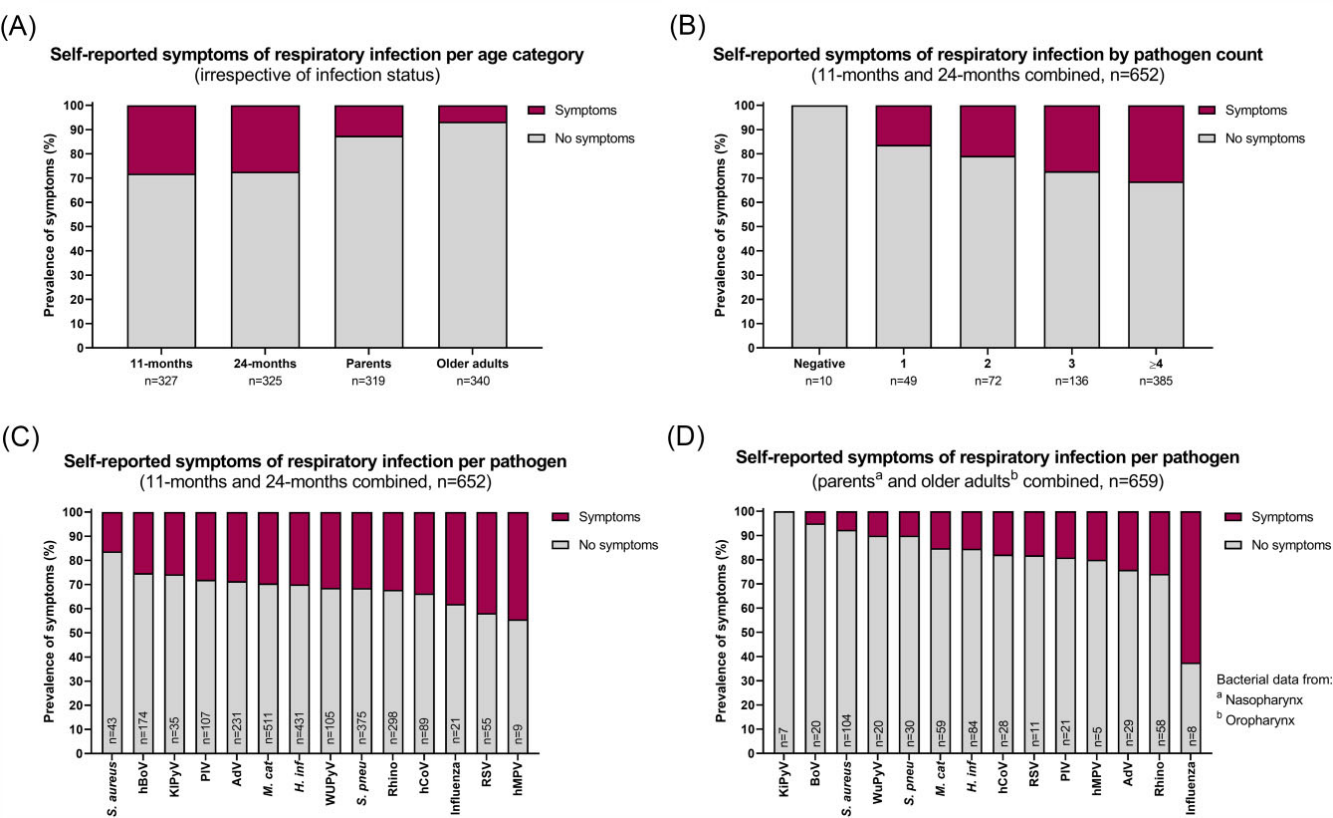
Occurrence of symptoms of respiratory infection in different age groups. Participants were asked whether, at the time of sampling, they experienced any symptoms of respiratory infection, e.g. cough, headache, or sneezing. (A) Overview of the prevalence of self-reported symptoms per age group irrespective of infection status. (B) Overview of the prevalence of self-reported symptoms by pathogen count for children (*n* = 652). (C) and (D) Overview of the prevalence of self-reported symptoms per pathogen for children (*n* = 652, C) and (older) adults (*n* = 659, D). Abbreviations: AdV, adenovirus; *H. inf, Haemophilus influenzae*; hBoV, human bocavirus; hCoV, human coronavirus; hMPV, human metapneumovirus; KIPyV, Karolinska Institute polyomavirus; *M. cat, Moraxella catarrhalis*; PIV, parainfluenza virus; Rhino, rhinovirus; RSV, respiratory syncytial virus; *S. aureus, Staphylococcus aureus; S. pneu, Streptococcus pneumoniae ;* and WUPyV, Washington University polyomavirus.

Next, we were interested to see to what extent the observed presence of respiratory pathogens is accompanied by the occurrence of symptoms related to respiratory infection. From the data collected during the interview it was apparent that, without taking infection status into account, children were significantly more likely to present with symptoms of respiratory infection than parents and older adults (*P* < .001, Fisher’s exact test, Fig. 2A). In addition, parents were significantly more likely to present with symptoms than older adults (*P* = .02, Fisher’s exact test). These findings are in line with the differences observed in pathogen prevalence between children, parents, and older adults.

Since our data revealed that pathogen codetections in children are very common, we were subsequently interested to see whether the number of pathogens per individual associated with the prevalence of symptoms of respiratory infection. Indeed, we observed an increasing trend in the prevalence of symptoms with increasing pathogen numbers (Fig. 2B), which was statistically significant when comparing the negative or 1 pathogen group to the ≥4 pathogens group (*P* = .04 and *P* = .03, respectively, Fisher’s exact test). Notably, it is clear that even in the group with the highest percentage of symptomatic individuals, i.e. children having ≥4 pathogens detected, the vast majority still presented as asymptomatic (69%). Whereas all of the children that were reported to show symptoms of respiratory infection (*n* = 181/181) tested positive for ≥1 pathogen, 15% of parents that reported to be symptomatic (*n* = 6/40) tested negative for all assessed pathogens in both nasopharyngeal and oropharyngeal swabs.

To obtain an indication of the association between the presence of certain pathogen species and the occurrence of symptoms of respiratory infection, we counted for each pathogen species the number of symptomatic and asymptomatic individuals. Because the manifestation of symptoms for specific pathogens might differ with age, we performed these analyses separately for children and adults. Notably, because of the low numbers of single infections in children, we did not correct for the presence of other pathogens in the same individuals. For example: of all children that tested positive for rhinovirus (and potentially other pathogens), 202 (68%) were asymptomatic and 96 (32%) were symptomatic at the time of sampling. For children, we found that infection with hMPV, RSV, and influenza virus was most frequently associated with symptoms of respiratory infection (Fig. 2C). In the case of parents and older adults, it appears that influenza virus, rhinovirus, and adenovirus infections were most frequently associated with symptoms (Fig. 2D). For both groups, the presence of *S. aureus*, hBoV, and KI polyomavirus was least often associated with symptoms of respiratory infection (Fig. 2C and D). Children appear more likely to be symptomatic than adults for all pathogens except influenza. Of note, for several pathogens the detection numbers are very low and these should therefore be interpreted with caution.

### Mucosal cytokine levels are generally higher in children compared to parents and associate with the presence of symptoms of respiratory infection

Since cytokines and chemokines are important mediators of the immune response and potential biomarkers or even determinants of disease severity, we wondered whether differences in their concentration relating to the presence of symptoms of respiratory infection could be observed in this cross-sectional cohort despite the absence of severe disease. To this end, we determined the concentration of proinflammatory chemokines, interleukins, interferons, and several cytotoxic effector molecules in a random selection of nasopharyngeal swab samples (*n* = 100 per group). As both viral and bacterial nasopharyngeal data were available for children and parents—but not for older adults—we focused on these groups for the cytokine analysis. Participant demographics for the selected subset can be found in Table 5.

**Table 5.** Participant characteristics, selection for cytokine analysis.

	11-months	24-months	Parents^a^
Sample size, *n*	104	102	100
Female sex, number (%)	53 (51.0)	55 (53.9)	55 (55.0)
Age in days, mean (range)	328 (311–362)	737 (707–758)	
Age in years, mean (range)			36 (23–51)
**Breastfeeding** ^b^			
Never, number (%)	18 (17.3)	24 (23.5)	
0 < 6 months, number (%)	43 (41.3)	39 (38.2)	
≥6 months, number (%)	43 (41.3)	39 (38.2)	
**Childcare**			
≥0.5 days/week, number (%)	79 (76.0)	80 (78.4)	
Duration in days/week, mean (range)^c^	2.2 (1–4)	2.1 (0.5–4)	
Group size, mean (range)	9.4 (0–18)	11 (2–25)	
**Siblings**			
≥1 siblings, number (%)	58 (55.8)	73 (71.6)	
Number of siblings, mean (range)^d^	1.2 (1–3)	1.5 (1–4)	
**BMI**			
Mean (range)			25.5 (17.2–44.1)
≥25, number (%)			44 (44.0)
**Influenza vaccination**			
2012/2013, number (%)			9 (9.0)

a Parents of the 24-month old children.

b Data are missing for one child in the 24 months group.

c For those children that attend childcare facilities.

d For those children that have siblings.

**Figure 3.**
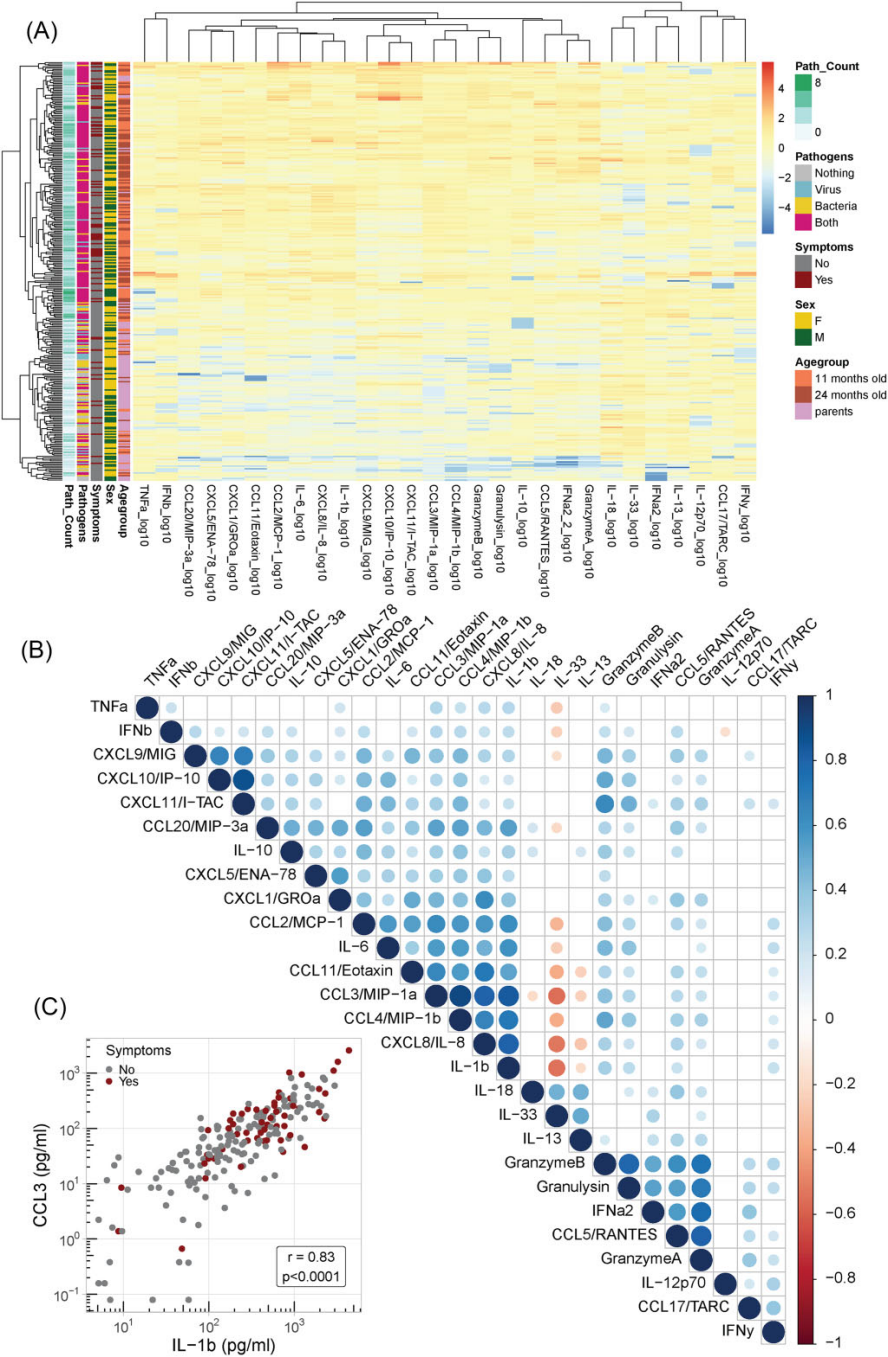
Heatmap and correlation matrix of mucosal cytokines determined in nasopharyngeal swabs. A multiplex immunoassay was used to determine the concentrations of several cytokines, chemokines, and effector molecules in nasopharyngeal swab samples of 11- and 24-month old children and parents (*n* = 100 per group). (A) A heatmap was produced using scaled data from all children and parents by performing unsupervised hierarchical clustering of both cytokines and participants based on Euclidean distance. Pathogen count (path_count), pathogen combination, presence of respiratory symptoms, sex, and age group of each participant are indicated on the left (a legend is present on the right side of the heatmap). (B) A correlation matrix was produced based on Spearman rank order correlation analysis of data from 11- and 24-month old children. Only correlations with a *P*-value below .02 are shown as dots, of which the size and color indicate the strength and direction of the correlation (also see legend on the right side of the matrix). (C) Example of a correlation plot for IL-1β and CCL3 using data from 11- and 24-month old children (*r* = 0.83, *P* < .0001, Spearman).

To obtain a general impression of the cytokine data we first generated a heatmap based on unsupervised hierarchical clustering of both cytokines and participants (Fig. 3A). Participant clustering basically resulted in a separation between children and parents, with some exceptions. Overall, children show higher mucosal cytokine levels than parents, in line with the observed differences in pathogen prevalence between these groups. IL-33 showed an opposite pattern with generally higher levels in parents compared to children. Clustering of cytokines resulted in grouping of functionally related molecules such as IL-1β/6/8 and CXCL9/10/11, suggesting that the measured cytokine levels were representative of biological events. For a more detailed overview of the various relations between each of the measured cytokines, we then prepared a correlation matrix based on the data of the children only (Fig. 3B), as the overall differences between children and parents might obscure other biologically relevant correlations. The correlation matrix revealed mostly positive correlations, except for IL-33 and IL-13 which showed a positive correlation with each other but a negative correlation with several other molecules including for example CCL3 and IL-1β. Similar to the heatmap clustering, several groups of functionally related molecules could be discerned within the correlation matrix, with some additional—mostly weaker—correlations between these groups. Fig. 3(C) shows an example of the strong positive correlation between IL-1β and CCL3 (Spearman rho = 0.83, *P* < .0001), within a group of several proinflammatory molecules.

**Figure 4.**
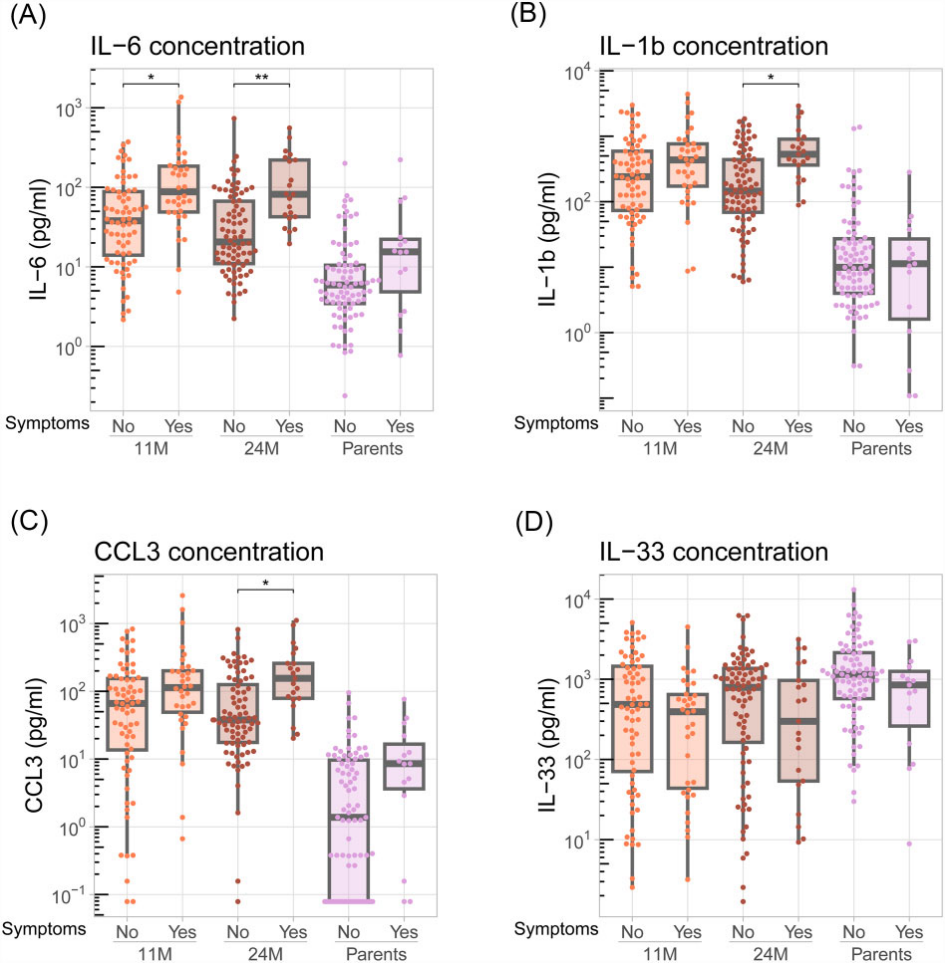
Cytokine concentrations in the nasopharyngeal mucosa of children and parents in the absence or presence of symptoms of respiratory infection. A multiplex immunoassay was used to determine the concentrations of among others IL-6 (A), IL-1β (B), CCL3 (C), and IL-33 (D) in nasopharyngeal swab samples of asymptomatic and symptomatic 11- and 24-month old children and parents (*n* = 100 per group). Samples below the limit of detection were set to 0.5 times the lowest detected concentration for analytical purposes. Boxplots depict median and quartiles. Statistical significance of the difference between symptomatic and asymptomatic individuals within age groups was assessed using a nonparametric Kruskal–Wallis test, followed by Dunn’s multiple comparisons test. * *P* < .05; ** *P* < .01.

Finally, we asked whether any of the measured cytokines associated with the presence of symptoms of respiratory infection in children and/or parents. Especially IL-6 showed a clear association with the presence of symptoms, which was statistically significant in 11- and 24-month old children (*P* < .05 and .01, respectively, Kruskall–Wallis, followed by Dunn’s multiple comparisons test) but not parents (Fig. 4A). Both IL-1β and CCL3 also showed an association with symptoms, but this was only statistically significant in 24-month old children (*P* < .05, Kruskall–Wallis; Fig. 4B and C). In line with its the negative correlation with IL-1β and CCL3 (Fig. 3B), IL-33 showed a trend toward lower levels in symptomatic compared to asymptomatic individuals, but this was not statistically significant (Fig. 4D). The raw cytokine data are available as supplementary material for additional in-depth analysis.

## Discussion

In this study, we show that respiratory pathogens are widely present in the generally healthy population. At least one respiratory pathogen was detected in >98% of 11- and 24-month-old children and in 26% of older adults, while parents showed an intermediate percentage of positivity (62%–69% depending on pharyngeal sampling site). Although only assessed at the time of sampling, it appears that a large part of these detections are not associated with clinical symptoms. Our analyses further confirm that childcare attendance and having siblings associate with significantly increased pathogen counts in young children. Finally, we show that the concentration of proinflammatory molecules such as IL-6, IL-1β, and CCL3 is increased in symptomatic compared to asymptomatic individuals.

The major strength of this study is that our sampling strategy did not depend on medically attended infections. For this reason, our data provide a snapshot of the prevalence of respiratory pathogens in the general population during the peak respiratory infection season, which is important information that helps to increase our understanding of microbial reservoirs and pathogen exposure. In addition, study participants included young children as well as parents and older adults. The inclusion of various subpopulations allowed for comparison of pathogen prevalence and species distribution across age groups. Finally, our analyses included an extensive diagnostic panel, consisting of both viruses and bacteria, and a broad range of cytokines and chemokines.

A limitation of our study is that it covered only one winter (2012/2013), and therefore does not account for variability in the circulation of pathogens within and between years. Of note, based on available information on national respiratory infection surveillance, this respiratory season was generally similar to other years (Teirlinck et al. 2015). Nevertheless, due to the nonpharmaceutical interventions implemented to contain the COVID-19 pandemic, circulation of a number of respiratory viruses, including influenza, RSV, hMPV, and PIV, has dramatically decreased in 2020/2021 (Kuitunen et al. 2020, Redlberger-Fritz et al. 2021, Tang et al. 2021, Yeoh et al. 2021). As the resumed circulation of these pathogens has shown atypical patterns in some cases, it remains to be seen how their circulation will take shape again in the future (Baker et al. 2020, Foley et al. 2021). Furthermore, the cross-sectional nature of the study does not allow for analysis of for example the duration of infection or the occurrence of repeated infections in the same individuals. Because of the lack of follow-up on the development of symptoms, we cannot draw firm conclusions on the percentage of asymptomatic infections. Probably for this reason, the percentage of individuals without symptoms in this study appears to be slightly higher than what has been found in earlier studies (Byington et al. 2015, Birger et al. 2018).

An important question in the context of infection prevention is which group of individuals form the main source of pathogen spread. Although we did not address this question directly in our study, our data supports the notion that, in general, young children are an important reservoir of respiratory pathogens. For example, childcare attendance, having siblings, and being a parent—all involving frequent exposure to young children—associate with an increased likelihood of being infected, which is in line with previous reports (Chu et al. 2013, Powell et al. 2022). In addition, the infection patterns observed in parents are in many ways intermediate between those in young children and older adults. The latter have less frequent contact with young children than parents, although additional (e.g. lifestyle or immunological) differences might also play a role. Furthermore, children appear to harbor more pathogen species per individual and to be symptomatic more often than adults, both of which potentially contribute to pathogen spread. For several pathogens, including bocavirus, adenovirus, and *S. pneumoniae*, their relative contribution appears to be higher in young children than in adults. In contrast, the relative contribution of endemic coronaviruses and *S. aureus* appears to be lower in young children compared to adults. These observations suggest that while young children may be important reservoirs for the spread of respiratory infections in general, their actual role likely depends on the specific pathogen in question. Additional data and modelling studies are needed to address these species-specific transmission dynamics.

The ubiquitous presence of both viral and bacterial respiratory pathogens, especially in very young children and often without clinical symptoms, begs the question whether and how their presence influences the development of local and systemic immunity. Many reports exist on the detrimental role of rhinovirus and RSV infections in the development and/or exacerbation of asthma, which is a chronic inflammatory disease of the lung (Busse et al. 2010, Lambrecht and Hammad 2015, Jartti and Gern 2017). However, (mild) infections might also play a beneficial role in the proper development of immunity, as has been suggested for the gut microbiome (Virgin 2014, Zheng et al. 2020). While these aspects of pathogen–host interactions are often difficult to investigate, the decrease in circulation of various respiratory pathogens due to nonpharmaceutical interventions during the COVID-19 pandemic might provide a unique window of opportunity for this. It would be interesting to examine whether the local and systemic immune response of children growing up under these conditions differs from that observed during regular circulation of these respiratory pathogens.

In addition to respiratory pathogen prevalence, we assessed the concentrations of numerous (proinflammatory) cytokines and chemokines in the nasopharyngeal mucosa of children and parents. Our data show that even in the absence of severe illness, marked differences in the levels of proinflammatory molecules can be observed that correlate with the presence of symptoms of respiratory infection. While the majority of cytokines that were assessed showed higher levels in children compared to adults, suggestive of a positive association with the presence of increased numbers of respiratory pathogens, the opposite was true for IL-33. This cytokine is a so-called “alarmin,” which is constitutively expressed by for example epithelial cells in the lung and released upon cellular necrosis (Johansson and McSorley 2019). IL-33 is mainly associated with type 2 immune responses and has been implicated in the development of asthma (Johansson and McSorley 2019). Because of the cross-sectional nature of our data, it remains unclear whether the observed differences in IL-33 levels precede pathogen acquisition or are in fact a result of infection. Interestingly, Robinson et al. (2018) have shown in a mouse model that influenza infection results in decreased *S. aureus*-induced IL-33 production, potentially leading to increased susceptibility to this pathogen. The observed lower levels of IL-33 in children would be in line with a scenario in which an initial (viral) infection increases the susceptibility to subsequent (bacterial) pathogen acquisition by means of IL-33 inhibition, thereby providing a possible explanation for the large numbers of pathogens detected in these younger individuals, but further research is needed.

With this study, we further establish the widespread nature of respiratory pathogens, both viral and bacterial, in the generally healthy population and their association with local cytokine and chemokine levels. These findings may guide further research that is needed to understand respiratory pathogen transmission dynamics and uncover the most appropriate strategies for disease prediction and prevention. Above all, it is clear that even in the absence of a pandemic, our respiratory tracts are habitually inhabited by a variety of pathogens which, fortunately, often go unnoticed.

## Supplementary Material

ftae010_Supplemental_Files
