# Supplementary material for: Prevalence of human respiratory pathogens and associated mucosal cytokine levels in young children and adults: a cross-sectional observational study in the Netherlands during the winter of 2012/2013
Source: Pathog Dis. 2024 May 7;82:ftae010. doi: 10.1093/femspd/ftae010 (PMC11132126; doi:10.1093/femspd/ftae010)
Supplement: ftae010_Supplemental_Files [file ftae010_supplemental_files.zip › Supplementary_figures.pdf]

Figure S1

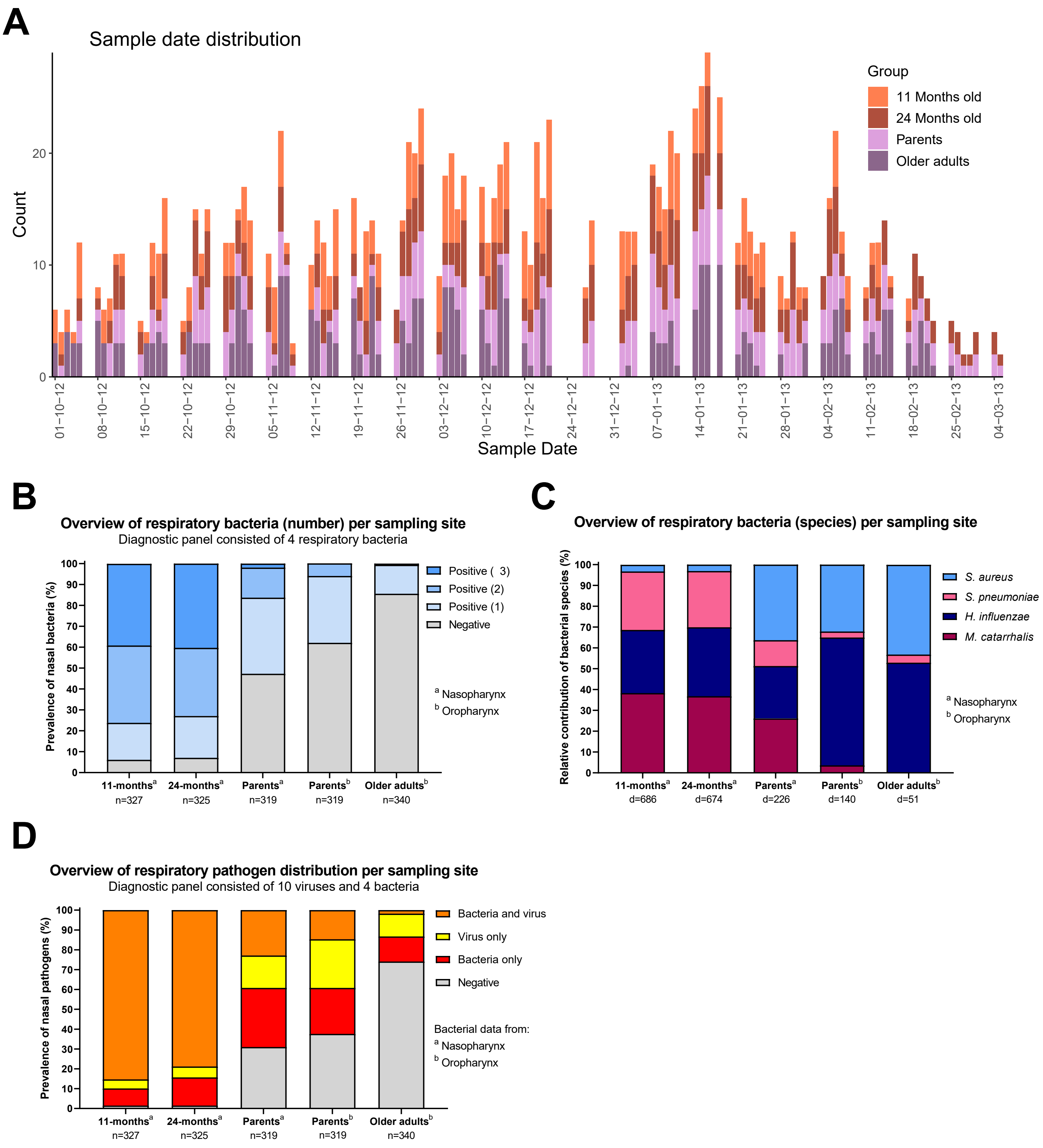

Figure S1. Overview of sampling dates and difference between nasopharyngeal and oropharyngeal swabs in bacterial prevalence. A) Overview of all individual sampling dates for 11- and 24-month old children, parents and older adults. **B)** The prevalence of respiratory bacteria in all age groups (n indicates group size) and **C)** the relative contribution of bacterial species (d indicates the total number of individual bacterium detections, a single participant can be included multiple times with the occurrence of co-detections) in different age groups. **D)** Overview of the distribution of viral infections and bacteria between individuals in different age groups. Groups consisted of 11-month old children (n=327), 24-month old children (n=325), parents (n=319), and older adults ≥60 years of age (n=340). Bacterial presence was determined in a) nasopharyngeal and/or b) oropharyngeal swabs depending on the age group.

Figure S2

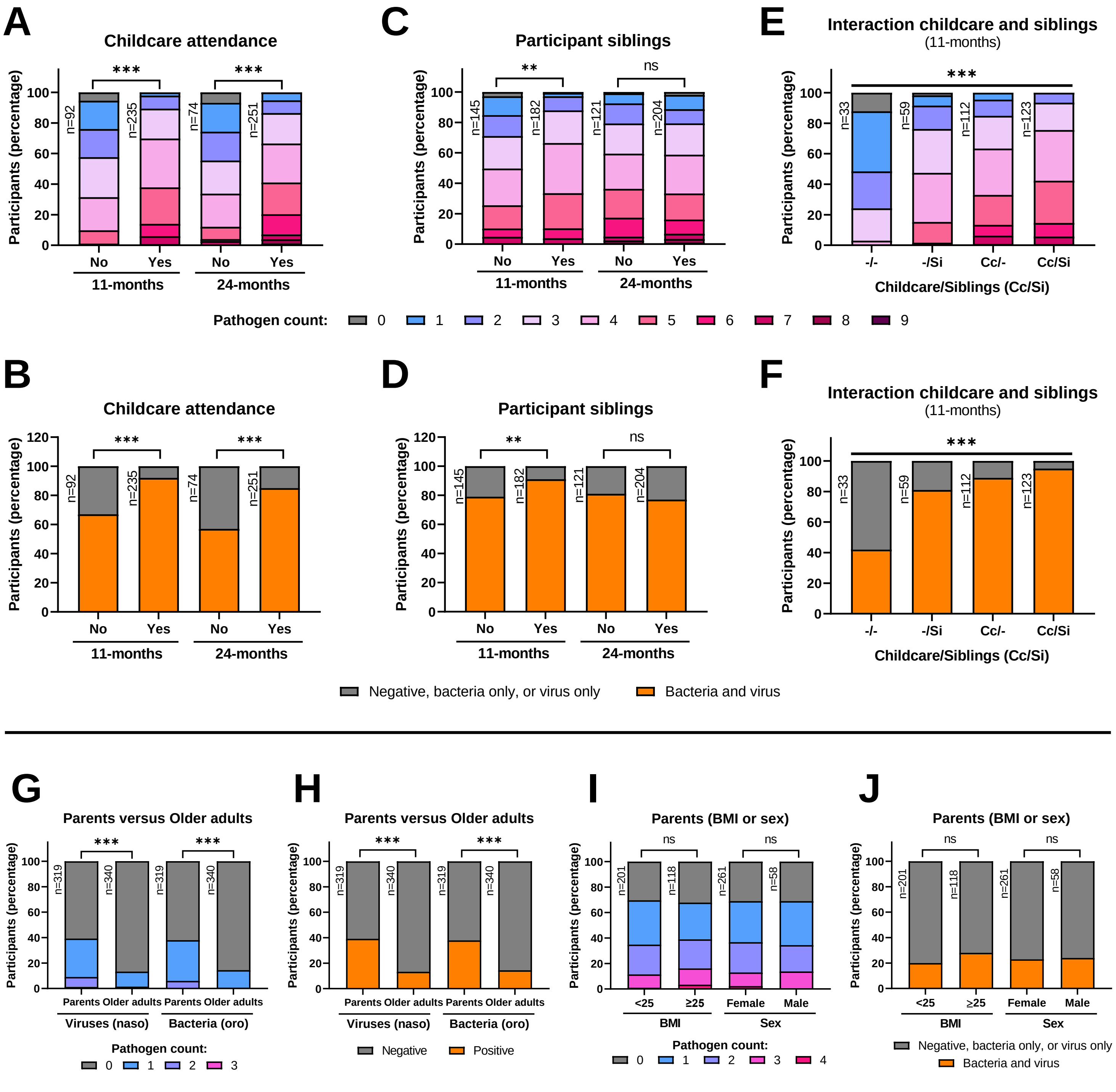

Figure S2. Risk factors for infection in children and adults. Association of childcare attendance with the number of pathogens (A) or bacterial/viral co-detection (B) in 11- and 24-month old children. Association of having siblings with the number of pathogens (C) or bacterial/viral co-detection (D) in 11- and 24-month old children. Interaction by childcare attendance and having siblings regarding the number of pathogens (E) or bacterial/viral co-detection (F) in 11-month old children. Difference between parents and older adults in the number of viruses/bacteria per individual (G) or the presence of viruses/bacteria (H). Association of sex and BMI with the number of pathogens (I) or bacterial/viral co-detection (J) in parents. Statistical significance was assessed using a nonparametric Kruskal–Wallis test, followed by Dunn's multiple comparisons test, for pathogen counts (A,C,I). Statistical significance of categorical data was assessed by Fisher's exact test (B,D,J). Interaction by childcare attendance and having siblings was assessed by Kruskal-Wallis test (E) or generalized Cochran-Mantel-Haenszel test (F), where the p-value was evaluated by permutation test. * P <0.05; ** P <0.01; *** P <0.001. Abbreviations: ns, not statistically significant; BMI, body-mass index.
